# Differences in close-work activities and optical axis length between only children and non-only children: a cross-sectional study

**DOI:** 10.1186/s12887-022-03586-7

**Published:** 2022-09-10

**Authors:** Yanhui Wang, Yaoyao Lin, Dandan Jiang, Linjie Liu, Shudan Lin, Juan He, Youping Liang, Bing Sun, Yanyan Chen

**Affiliations:** 1 Wenzhou Medical University, Zhejiang Wenzhou, China; 2 The Eye Hospital, Wenzhou Medical University, 270 Xueyuan Road, Wenzhou, 325027 Zhejiang China

**Keywords:** Myopia, School-aged children, Few children family, Optical axial length, Close-work activities

## Abstract

**Background:**

This study aimed to investigate the differences in optical axial length and close-work activities between only children and children with siblings in Wenzhou.

**Methods:**

This was a cross-sectional population-based study. In total, 2913 school-aged children and their parents in Wenzhou were included as study subjects from April to May 2021. Data regarding the optical axial length, spherical equivalent refraction, number of children in a family, parental myopia, and close-work activities were collected through eye examinations and questionnaires. A multivariable logistic regression was used to analyze the association between the number of children in a family and optical axial length.

**Results:**

The children were aged 9.80 ± 3.41 years. The overall percentage of children with an axial length > 24 mm was 38.9%, 44.5% in only children and 35.6% in multiples. The multivariable logistic regression analysis showed that the odds of having an AL > 24 mm were 1.24 times higher in only children than in multiples (OR: 1.24, 95% CI: 1.025–1.480, *P* = 0.028). Only children were 1.331 times more likely to perform homework > 1 h on weekends than multiples (OR: 1.331, 95% CI: 1.049–1.688, *P* = 0.019). Only children in upper grades were 1.543 times more likely to perform homework > 1 h on weekends than multiples (OR: 1.543, 95% CI: 1.065–2.235, *P* = 0.025). Boys who were only children were more likely to attend three or more extracurricular classes for academic subjects than multiples (OR: 1.224, 95% CI: 1.011–1.562, *P* = 0.004).

**Conclusions:**

Being an only child may be associated with a higher risk of myopia and higher odds of close-work behaviors. Only children, especially those in upper grades, are more likely to spend more time on homework than their peers who are multiples. Only children, especially boys, are more likely to attend extracurricular classes in academic subjects.

**Trial registration:**

This trial is registered as ChiCTR1900020584 at www.Chictr.org.cn.

## Background

Myopia is a worldwide public health problem and a chronic disease [1, 2]. The global prevalence of myopia was estimated to increase from 28.3% to 34.0% from 2010–2020, with an increase of approximately 20% from the baseline prevalence [3, 4]. Myopia occurs in young children; the most common type of myopia in children is axial myopia [5]. The axial length (AL) is the most important determinant of refractive error of the AL and reflects the lens thickness, anterior chamber depth and vitreous chamber depth [6]. Growing evidence suggests that AL better predicts the risk of developing myopia and high myopia [7].

To effectively control the incidence of myopia, it is important to identify the population at risk and adopt effective measures. The occurrence of myopia is closely related to genetic and environmental exposures, and the family structure is considered a potential risk factor [8, 9]. Subreplacement fertility is a cause of the changing family structure [10]. Subreplacement fertility, or a low fertility rate (less than 2 births per woman), has persisted in developed countries in recent decades and is spreading globally [11]. Nearly half of the population worldwide currently lives in countries with similar or lower fertility and replacement rates [12]. As of 2015, the number of one-child families in China was 224.6 million [13]. China fully opened the "two-child" policy in 2016, but there are still many one-child families in the country [14].

The impact of the family structure on children's health has been of great concern in recent years [15]. Compared to families with multiples, one-child families have a concentration of family resources and access to more protective health-promoting factors, such as more time for parental companionship and supervision [16]. However, changes in the family structure have also introduced many challenges to the healthy development of only children [17, 18]. A check-up of 33,194 high school students showed a myopia rate of 83.5% among only children and 76.5% among multiples, suggesting that only children may have a high prevalence of myopia, and the acceleration of myopia in only children among high school students may be the result of more educational pressure on only children [19]. In recent years, the prevalence of myopia among elementary school students in China has been increasing yearly, and the age of myopia onset is decreasing [4, 20]. Whether the change in the family structure due to a low fertility rate, i.e., the number of children in a family, has an effect on the development of myopia in children is unclear.

The commonly accepted myopia risk behaviors in existing studies include close-work activities and outdoor activities. Close-work activities include homework, reading, watching TV, and using electronic devices, such as cell phones and tablets, all of which increase the risk of myopia. However, knowledge regarding whether there are significant differences in close-work activities between only children and multiples is limited. We investigated children's AL, close-work activities, sleep, participation in extracurricular classes for academic subjects, and parental myopia. Our study aims to provide a scientific basis for the development of myopia prevention and control strategies by comparing the risk of myopia in only children and multiples and assessing the association between the condition of only children and close-work activities.

## Methods

### Design and subjects

This study was a school-based cross-sectional study called the Wenzhou Epidemiology of Refraction Error (WERE). A stratified random sampling method was used to select three of the 64 elementary schools in the Lucheng District of Wenzhou in southeastern China. The three schools share similar campus cultures, teaching levels, and community socioeconomic statuses. All students in grades 1 to 6 and one of their parents were surveyed from April to May 2021. In total, 3514 subjects who provided informed consent were included in this study, which involved height and weight measurements, eye examinations and questionnaires. In total, 2913 students were enrolled in this study; 601 students were excluded from the analysis due to logical errors or missing responses on the questionnaire > 5% (*n* = 568), comorbid eye diseases or the inability to cooperate during the examination (*n* = 33). This study was approved by the Ethics Committee of the Affiliated Optometric Hospital of Wenzhou Medical University (Approval Number: KYK【2018】49).

### Anthropometric measurements

The AL was measured using an optical coherent biomechanometer (Lenstar 900 Optical Biometer; Haag-Streit, Koeniz, Switzerland), and refractive error (noncycloplegic) was measured using a Topcon RM8900 autorefractor (Topcon Co., Tokyo, Japan), with each eye being measured at least three times to determine the average value. Spherical equivalent refraction (SER) = sphere + cylinder/2. Myopia is defined as SER ≤ -0.5D [4]. Weight and height were measured to obtain the body mass index (BMI). Since the equivalent spherical vista of both eyes is highly correlated with the parameters of the eyeball, all test results were statistically analyzed based on the right eye.

### Questionnaire

The children's questionnaire was a self-administered questionnaire by the subject group with proven reliability and validity and a Cronbach's alpha coefficient of 0.82 [21]. The children's survey included general questions regarding the student (sex and grades), close-work activities (weekday and weekend averages of time spent performing homework per day, time spent using a cell phone per day, time spent using a computer per day, and time spent performing outdoor activities per day) and sleep (average amount of sleep per night over the last month). The content of the parental report included the parent's myopia (both sides were myopic; one side was myopic; neither was myopic; or unclear) and the child's participation in extracurricular classes for academic subjects (the number of subjects studied in additional classes). The number of children in a family was determined by a response to the following question on the parental survey: "Is the child an only child?" If the parents answered yes, the child was classified as an only child; otherwise, the child was classified as a multiple. A high parental education level was defined as possessing a bachelor's degree or higher.

### Quality control

All randomization processes were carried out by a staff member who was not involved in the survey. The investigation process was formulated before the investigation, and the investigators were trained in a standardized manner. All survey sites used the same process during implementation. The questionnaire was distributed to students, who completed it within the time specified in a designated classroom. The questionnaire was collected immediately, and the investigators checked for omissions and asked the subjects to complete any missing items. The parental questionnaires were taken home by the students and given to their guardians to complete. Questionnaires with illogical responses were eliminated before the data were analyzed. All questionnaire data were double-entered to ensure completeness and accuracy.

### Statistical analysis

The primary outcome of this study was AL. The study's secondary indicator was myopia. The main exposure factor was whether the child was an only child. All data were analyzed using the statistical software SPSS 20.0 (Statistics 20.0, SPSS, IBM, Armonk, NY, USA). The Kolmogorov–Smirnov test was used to observe whether the continuous variables were normally distributed; continuous variables that did not conform to a normal distribution are expressed as medians (upper and lower quartiles), and the categorical variables are expressed as composition ratios/rates. Based on previous studies, this study divided the grades into the following two categories: upper grades and lower grades [22]. A χ^2^ test and Mann–Whitney U test were used to analyze the differences in the categorical or continuous variables between the only children and multiples. A multivariable logistic regression was used to assess the odds ratio (OR) of the association between being an only child in a family and AL, myopia, and close-work activities. Confounders, including gender, grade, BMI, parental myopia, and parental education level, were adjusted. Two-tailed *P*-values were used in all analyses, and the results were considered significantly different if the *P*-value was less than 0.05.

## Results

### Characteristics of the study population by only children and multiples

In total, 2913 children and their parents (1071 only children) were recruited, with an average age of 9.8 ± 3.41 years; 1557 of the children were boys (53.5%), and 1424 were senior students (48.9%) (Table 1). The proportion of boys who were only children was higher than that of girls in the study population (*P* < 0.001). The average AL of the study subjects was 23.69 (22.99, 24.46) mm. In addition, compared to the multiples, a higher percentage of only children performed > 1 h of homework on weekdays (86.3% vs. 83.5%), > 1 h of homework on weekends (87.2% vs. 83.9%) and ≥ 3 extracurricular classes for academic subjects (33.5% vs. 28.5%), but a lower percentage obtained > 9 h of sleep per night (11.4% vs. 14%).

**Table 1:** Descriptive characteristics of the study population

Variables	Total (*N* = 2913)	Only child (*N* = 1071)	Non-only child (*N* = 1842)	*P-value*
**Sex, n (%)**				
Boys	1557 (53.5)	631 (58.9)	926 (50.3)	< 0.001
Girls	1356 (46.5)	440 (41.1)	916 (49.7)	
**Grade, n (%)**				
Lower grades (Grade 1 to 3)	1489 (51.1)	530 (49.5)	959 (52.1)	0.180
Upper grades (Grade 4 to 6)	1424 (48.9)	541 (50.5)	883 (47.9)	
BMI	16.72 (15.22, 18.92)	17.13 (15.38, 19.18)	16.49 (15.14, 18.74)	< 0.001
Height	139.00 (130.00, 149.35)	140.00 (130.50,150.00)	138,50 (130.00,149.00)	0.018
**Parental myopia, n (%)**				
None	681 (23.4)	147 (14.5)	534 (31.0)	< 0.001
One	1023(35.1)	392 (38.7)	631 (36.6)	
Both	1034 (35.5)	474 (46.2)	560 (31.9)	
Unclear	175 (6.0)	58 (5.4)	117 (6.4)	
Maternal education level**, n (%)**				
≤ High school	987 (33.9)	205 (19.2)	782 (42.5)	< 0.001
≥ Undergraduate	1926 (66.1)	868 (80.8)	1058 (57.5)	
Paternal education level**, n (%)**				
≤ High school	1162 (39.9)	298 (27.6)	864 (47.0)	< 0.001
≥ Undergraduate	1751 (60.1)	776 (72.4)	975 (53.0)	
**Close-work activities, n (%)**				
Homework on weekdays > 1 h	2423 (84.5)	805 (86.3)	1518 (83.5)	0.045
Homework on weekends > 1 h	2414 (85.1)	907 (87.2)	1507 (83.9)	0.017
Cell phone use on weekdays > 1 h	817 (28.7)	278 (26.7)	539 (29.8)	0.075
Cell phone use on weekends > 1 h	1232 (43.9)	450 (43.8)	782 (44)	0.922
Computer use on weekdays > 1 h	369 (13)	135 (13)	234 (12.9)	0.965
Computer use on weekends > 1 h	536 (19.1)	188 (18.4)	348 (19.5)	0.461
Outdoor activities on weekdays > 2 h	974 (34.4)	376 (36.4)	598 (33.2)	0.088
Outdoor activities on weekends > 2 h	1528 (54.4)	574 (55.9)	954 (53.5)	0.211
Sleep every night > 9 h	368 (13.1)	118 (11.4)	250 (14)	0.046
Attend learning classes ≥ 3	859 (30.3)	347 (33.3)	512 (28.5)	0.008

### Comparison of AL between only children and multiples by sex and grade

**Fig. 1:**
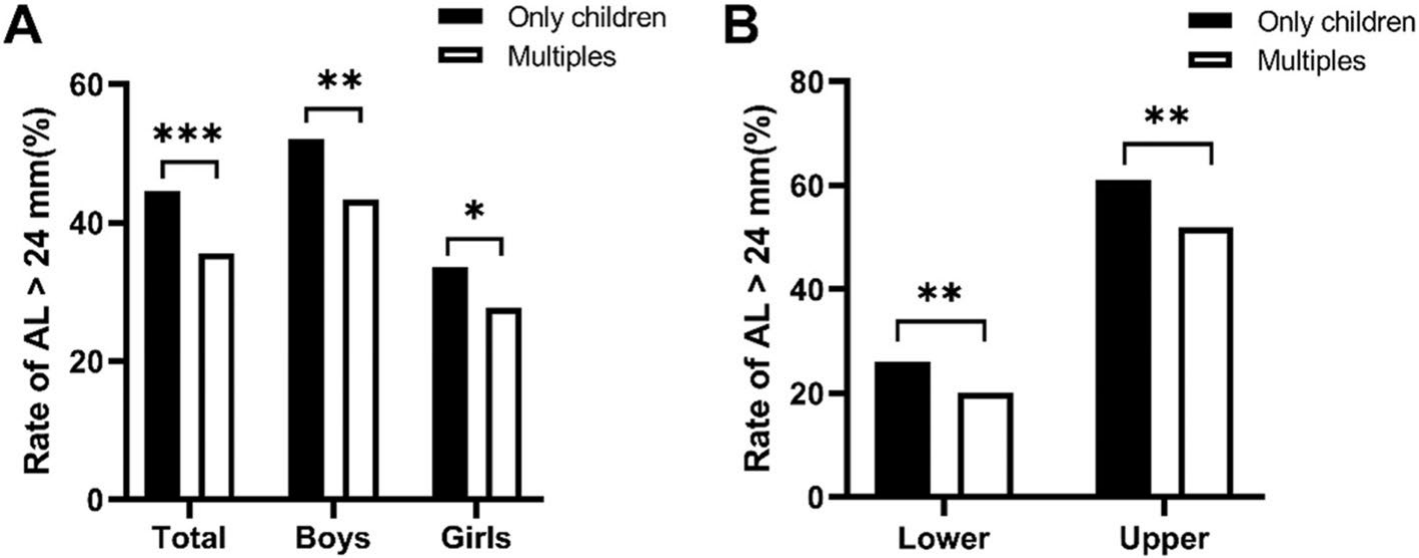
Comparison of the AL in only children vs. multiples by sex and grade. Comparison of the rates of AL > 24 mm in only children and multiples overall and by sex (**A**). Comparison of the rate of AL > 24 mm by grade in only children and multiples (**B**). *, *P* < 0.05; **, *P* < 0.01; ***P < 0.001

The participants were grouped by whether the AL was greater than 24 mm (1 = AL ≤ 24 mm, 2 = AL > 24 mm). Figure 1 shows the odds of having an AL > 24 mm in only children and multiples stratified by sex and grade. The overall rate of AL > 24 mm in the study population was 38.9%. In this study, the rate of AL > 24 mm was found to be 44.5% in the only children and 35.6% in the multiples (*P* < 0.01). In the subgroup analysis by sex and grade, the rate of AL > 24 mm was higher in the only children than in the multiples; specifically, the rate of AL > 24 mm was the highest in the upper grades in the only children (61.9%) and the lowest in the lower grades in the multiples (20.3%).

### The relationship among the number of children in a family, AL and close-work activities

The associations among the number of children in a family, the growth of AL and close-work activities were assessed using multivariable logistic regression models (Table 2). Among all participants, AL was associated with being an only child, and the odds of having an AL > 24 mm were 1.24 times higher in the only children than in the multiples (OR: 1.24, 95% CI: 1.025–1.480, *P* = 0.028). In addition, after adjusting for other confounding factors, only children were associated with weekend homework time, and only children were associated with the odds of writing > 1 h on weekends (OR: 1.331, 95% CI: 1.049–1.688, *P* = 0.019). A chi-square test found a longer AL (*P* < 0.05) and a greater risk of myopia (*P* < 0.05) in the children who attended 3 or more extracurricular classes for academic subjects.

**Table 2:** The association among the number of children in a family, the growth of AL and close-work activities

	**Model 1**		**Model 2**		**Model 3**	
**Outcome variables**	**OR (95% CI)**	***P-value***	**OR (95% CI)**	***P-value***	**OR (95% CI)**	***P-value***
AL > 24 mm	**1.452(1.245, 1.693)**	** < 0.001**	**1.370(1.156,1.625)**	** < 0.001**	**1.240(1.025,1.480)**	**0.028**
Myopia	1.029(0.884, 1.196)	0.714	0.981(0.834, 1.154)	0.819	0.915(0.766, 1.092)	0.324
Homework on weekdays > 1 h	**1.246(1.005, 1.545)**	** < 0.045**	1.223(0.982,1.524)	0.072	1.058(0.835,1.340)	0.640
Homework on weekends > 1 h	**1.308(1.048, 1.632)**	**0.017**	**1.304(1.042,1.633)**	**0.021**	**1.331(1.049,1.688)**	**0.019**
Sleep every night > 9 h	**0.788(0.624, 0.996)**	**0.046**	0.815(0.641,1.036)	0.095	0.947(0.731,1.227)	0.681
Extracurricular classes for academic subjects ≥ 3	**1.249(1.060, 1.473)**	**0.008**	**1.240(1.048,1.468)**	**0.012**	1.068(0.901,1.355)	0.182

^*^Bold text indicates a significant difference at *P* < 0.05. Model 1 was a binary regression model. Model 2 was adjusted for sex and grade. Model 3 was adjusted for all parameters in Model 2 and BMI, parental myopia and parental education level. *OR* Odds ratios, *CI* Confidence interval

### Relationship among the number of children in a family, AL and close-work activities by sex and grade

Table 3 shows that the odds of senior students performing > 1 h of homework on the weekend are positively correlated with only children (OR: 1.543, 95% CI: 1.065–2.235, *P* = 0.025). The only children in lower grades had a positive correlation with performing homework > 1 h on weekdays (OR: 1.277, 95% CI: 1.012–1.647, *P* = 0.028). In addition, boys who were only children were more likely to attend three or more extracurricular classes for academic subjects (OR: 1.224, 95% CI: 1.011–1.562, *P* = 0.004). The only children in lower grades were more likely to attend three or more extracurricular classes for academic subjects (OR: 1.424, 95% CI: 1.095–1.852, *P* = 0.003).

**Table 3:** The association among the number of children in a family, the growth of AL and close-work activities in different subgroups

	**Sex**		**Grade**	
**Outcome variables**	**Boys**	**Girls**	**Lower**	**Upper**
AL > 24 mm	**1.229(1.042,1.607)**	0.974(0.723,1.311)	1.144(0.867,1.508)	**1.341(1.069,1.723)**
Myopia	0.873(0.704,1.083)	0.946(0.723,1.238)	0.811(0.630,1.044)	1.019(0.791,1.313)
Homework on weekdays > 1 h	1.009(0.736,1.384)	1.109(0.774,1.588)	**1.277(1.012,1.647)**	0.843(0.572,1.242)
Homework on weekends > 1 h	1.318(0.966,1.800)	1.3540.935,1.961)	1.194(0.874,1.630)	**1.543(1.065,2.235)**
Sleep every night > 9 h	0.926(0.650,1.319)	0.974(0.666,1.426)	0.838(0.616,1.142)	1.256(0.777,2.028)
Extracurricular classes for academic subjects ≥ 3	**1.224(1.011,1.562)**	0.857(0.654,1.123)	**1.424(1.095,1.852)**	0.784(0.611,1.007)

^*^Bold text indicates a significant difference at *P* < 0.05. The model was adjusted for sex, grade, BMI, parental myopia and parental education level

## Discussion

In this cross-sectional study, we investigated the association among the number of children in a family, the growth of AL and close-work activities in children. The prevalence of myopia among the only children and multiples was 46.7% and 46.0%, respectively. The results showed that AL was longer in only children than children with siblings, suggesting that only children may be at a higher risk for myopia. The only children were more likely to spend > 1 h on homework on weekends, especially those in the upper grades. The only children were more likely to attend three or more extracurricular classes for academic subjects, especially the boys in the lower grades.

Our study shows that the odds of having an AL > 24 mm in only children were 44.5%, which was 9% higher than the odds of having an AL > 24 mm in the multiples. In a study conducted to determine the incidence of myopia and high myopia based on refraction without cycloplegia among children, the correlation between AL and SER was strong in both the elementary cohort and the middle school cohort [23]. Thus, AL, as an objective indicator of myopia that can indicate a high risk of myopia in only children. A cross-sectional study of 7119 junior high school students from the China Education Tracking Survey also showed that the prevalence of myopia was 71.71% among only children and 61.83% among multiples [24]. The results of our study differed likely because middle school students have more severe visual acuity than elementary school students. The results of another population-based cohort study showed that from 1990–2005, the proportion of only children among junior high school students increased from 5% to 42.7%, and the prevalence of myopia increased by 3.43% during the same period. This result suggests that only children may have an increased risk of myopia by 15 percentage points [8]. The high and increasing prevalence of myopia may be due to increased educational pressure and family environment [25]. However, these studies used SE (spherical equivalent) to assess the myopia status, and few focused on the growth of the AL, which has been shown in previous studies to significantly improve the sensitivity of early myopia prediction in children and adolescents [7] and may be a better predictor of axial myopia [26] and high myopia [27]. AL can also induce the development of myopia through retinal defocusing mechanisms or inflammatory mechanisms [4, 28]. This study used an objective indicator of AL to assess the association between myopia and the only child status to reduce measurement error due to nondilated pupils.

Although previous studies have demonstrated an association between the number of children in a family and the risk of myopia, the underlying cause remains unclear. The results of this survey show that only children were more likely to spend > 1 h/d on homework than multiples (OR: 1.328, 95% CI 1.052–1.678, *P* = 0.017). Previous studies have shown that only children spend 0.6 h more time per day on homework than multiples [24], which is consistent with the results of our research investigation. A study investigating the prevalence of myopia in Amman children found that the prevalence of myopia increased by 24% for each additional hour spent writing at close range after school [29]. Prolonged proximity work can lead to lagging eye regulation such that objects are imaged behind the retina, and the eye grows toward defocus in a long-term hyperopic out-of-focus state, with compensatory growth of the AL, leading to the development of myopia [30]. Our study found that only children tend to have more writing time but spend less time using electronics. Wang et al. [24] showed that there is a significant mediating role of close-work activities in the effect of the only child status on myopia prevalence likely because the parents of only children tend to provide an environment in which children have more writing time and less use of electronics. Previous studies have demonstrated this result [31]. It is recommended that children’s close-work activities be restricted, that they use their eyes reasonably and that they increase outdoor activity time.

This study also found that a higher percentage of only children attended three or more extracurricular classes for academic subjects than the multiples (OR: 1.424, 95% CI: 1.095–1.852 *P* = 0.012). Differences in the family structure lead to differences in children's education spending across families [32]. A previous study investigating urban families' education consumption also found that urban families with one child spend much more than families with multiple children [33]. Notably, after adjusting for other factors, only children in the lower grades are disproportionately enrolled in subject tutoring classes likely because Chinese education is more competitive, education is an important concern for parents of only children, and parents want their children to succeed early; thus, only children are enrolled in extracurricular classes early. A cohort study in Taipei found that people who spent ≥ 5 h per week in after-school tutoring classes had a 1.12 times higher risk of developing myopia [34]. Our study also found that children's participation in extracurricular tuition classes was associated with AL. It is recommended that parents of only children make better use of their free time by accompanying their children to exercise outdoors and reduce the time their children spend in close work.

In addition, this study found that male only children attended extracurricular classes more often than male non-only children (OR: 1.224, 95% CI: 1.011–1.562, *P* = 0.004). Previous studies have shown differences in parents' educational expectations for their only child and non-only child, with only child families having "higher" educational expectations for their children than non-only child families [35]. Statistics show that only child families place more emphasis on their children's participation in interest classes as a way to improve their overall quality [8, 36]. One study showed that among male students, the level of family supervision and education was significantly higher among parents of only children than among parents of non-only children [37]. Although the educational expectations of one-child families are not influenced by the sex of their children, there is still a preference for boys in the educational expectations of families with multiples [38]. Our findings also showed that only children in upper grades were more likely to spend > 1 h on homework than non-only children in upper grades (OR: 1.543, 95% CI: 1.065–2.235, *P* = 0.025). The high concentration of resources in one-child families and high parental investment in education can cause only children to feel more pressure to perform academically, especially as they progress through grades. Studies have shown that families with only children are more likely to supervise their children's studies, home education is more strictly managed, and only children spend more time performing homework and attending tutoring classes than non-only children [8, 39, 40]. Children in upper grades take more subjects, have heavier workloads, and may also spend more time on close-work activities than children in lower grades. It is recommended that schools and parents give their children more opportunities to engage in outdoor activities to reduce the pressure and burden of schoolwork, and boys are encouraged to participate in more outdoor training.

Our findings have significant implications for myopia prevention and control policies. First, only children in China may be a priority population for future myopia interventions, and health education measures at the family and school levels should guide them to adopt healthy close-work activities at an early stage. Second, as education becomes increasingly competitive and demands are placed on the overall quality of children’s education, children continue to be exposed to increasingly severe myopia, making it important to assess the impact of the only child status on myopia trends in China. In addition, the visual health of only children is an issue that needs attention in the future, and our study can provide a reference for countries with low fertility rates.

The present study also has certain limitations. First, there may have been recall bias in collecting information through questionnaires. Second, our study was a cross-sectional study with possible confounding, selection bias, and reverse causality. We did not measure head circumference in our study, which may be related to AL, but we considered the BMI and, thus, controlled for correlates of height and growth development. Additional longitudinal cohort studies and multicenter studies are needed to further clarify the association and factors influencing the prevalence of myopia among only children.

## Conclusion

In summary, this study shows that the risk of myopia is higher among children from one-child families. An only child is more likely to engage in close-work activities, which may be related to more parental attention and higher educational expectations, especially among boys. This finding suggests that we should pay more attention to this high-risk group in future myopia prevention and control. We urge schools and parents to reduce the academic burden of school-aged children and pay more attention to the visual health of only children.

## Data Availability

The datasets analyzed in this study are available from the corresponding author (Yanyan Chen, wzcyymail@163.com) upon reasonable request.

## Abbreviations

BMI: Body mass index
AL: Axial length
OR: Odds ratio
CI: Confidence interval
SE: Spherical equivalent
SER: Spherical equivalent refraction
